# DFT study, and natural bond orbital (NBO) population analysis of 2-(2-Hydroxyphenyl)-1-azaazulene tautomers and their mercapto analouges

**DOI:** 10.1038/s41598-023-50660-w

**Published:** 2024-01-02

**Authors:** Shimaa Abdel Halim, Asmaa B. El-Meligy, Ahmed M. El-Nahas, Safinaz H. El-Demerdash

**Affiliations:** 1 Chemistry Department, Faculty of Education, Ain Shams University, Cairo, 1171 Egypt; 2 Chemistry Department, Faculty of Science, Menoufia University, Shebin El-Kom, 32512 Egypt

**Keywords:** Chemistry, Physics

## Abstract

Theoretical research on the **keto-enol** tautomerization of 2-(2-Hydroxyphenyl)-1-aza azulene (2HPhAZ) and its **thiol-thione (**2MPhAZ) analouge has been performed using the density functional B3LYP method with the 6–311 +  + G(2d,2p) basis set in gas and ethanol phases. The findings of the MO computation on the energy scale and the prediction of the frontier molecular orbital (FMO) energies demonstrate that the tautomeric structures exist in a static mixture in the ground state, with the **enol** and **thiol** structure being more stable than the **keto** and **thione** structures in gas phase. The ethanol solvent causes some reordering of the relative stability of 2HPhAZ and 2MPhAZ conformers. The geometries created at the B3LYP/6–311 +  + G(2d,2p) level of theory were used for NBO analysis. In the tautomerization of 2HPhAZ and its mercapto analogue 2-(2-Mercaptophenyl)-1-azaazulene (2MPhAZ), it has been found that the O(S)-C sigma bond is weak due to *n*_*O*(*S*)_ **—> **σ*_C25-O26(S26)_ and *n*_*O*(*S*)_ **—> **σ*_C15-N16_ delocalization. It is also noted that the resulting p character of the corresponding oxygen (sulfur) natural hybrid orbital (NHO) of σ_*O(S*)-*C*_ bond orbital is related to the decreased occupancy of the localized σ_*O(S*)-*C*_ orbital in the idealized Lewis structure or the increased occupancy of σ*_O(S)-*C*_ of the non-Lewis orbital and their subsequent impact on molecular stability and geometry (bond lengths) in gas phase and ethanol. Additionally, the energy of charge transfer decreases as the potential rotamers' Hammett constants (R1–R3 for O(S) atoms) increase. The partial charge distribution on the skeleton atoms demonstrates that the intra- and intermolecular interactions can be significantly influenced by the electrostatic attraction or repulsion between atoms. Lastly, the currently applied NBO-based HB strength indicator enables a fair prediction of the frequency of the proton donor NH stretching mode, but this simple picture is hidden by abundant hype conjugative effects.

## Introduction

**Figure 1:**
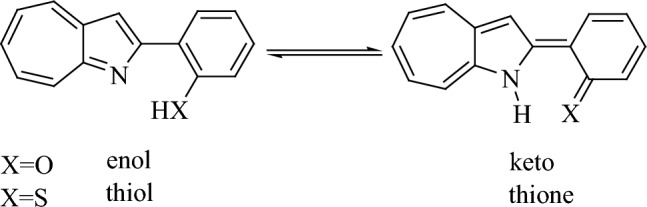
The tautomeric structure (enolimines/thiol and ketoenamines/thione form) of 2HPhAZ and 2MPhAZ.

Azaazulenes, an azulene heterocyclic analogue (Fig. 1), have attracted concern due to their chemical and physical properties and their biological activity^1,2^ as anticancer agents^3^. 1-azaazulenes are the most stable of the several azaazulene structures. When nitrogen and oxygen (sulphur) atoms in the heterocyclic ring change hydrogen atoms, tautomers are produced. The proton transfer and hydrogen bonding of the hydrogen atom are significant properties in chemistry. As a result, equilibrium between (enolimines/thiol and ketoenamines/thione forms) of 2HPhAZ and 2MPhAZ tautomeric forms is expected (Fig. 1).

Understanding the relative stabilities of tautomeric forms and their mutual conversion is a crucial topic from the standpoint of structural chemistry. Oda et al.^4^ demonstrated that spectroscopic and X-ray crystallographic investigations supported the structure of 2OHPhAZ. A reasonably substantial absorption in the visible light spectrum, as well as the X-ray diffraction data^4^, support the presence of its aromatic and coplanar nature and an intramolecular hydrogen bond. The stability of 2OHPhAZ, the keto tautomer (2OPhAZ), and various rotamers (2OHPhAZ-R1:R3) in the gas phase and ethanol was the subject of DFT research by El-Meligy et al.^5^. Furthermore, to the best of our experience, we have not found any analysis of the nature of the bonding in the tautomerization of 2-(2-Hydroxyphenyl)-1-azaazulene (2HPhAZ) and its mercapto analogue 2-(2-Mercaptophenyl)-1-azaazulene (2MPhAZ).

The study of covalence and hybridization effects in polyatomic wave functions led to the development of the natural bond orbital (NBO) analysis method. The work of Foster and Winhold^6^ was expanded by Reed et al.^7^, who utilized NBO analysis that showed mainly H-bonded and other strongly bound van der Waals complexes. The "natural Lewis's structure" has filled NBOs that are excellently suited to describing covalency effects in molecules^7^. However, the general conversion to NBOs also produces orbitals that are vacant in the official Lewis structure and can be utilized to characterize non-covalent effects.

The terms filled and unfilled orbitals of the formal Lewis structure are denoted, respectively, by the symbols σ and σ*, though the former orbitals may be core orbitals (*CR*), lone pairs (*LP*), *σ* or *π* bonds (*σ*, *π*), and so forth, and the latter may be *σ* or *π* anti-bonds (σ*, π*), extra valence shell Rydberg (RY*) orbitals.

The NBO analysis focuses on the availability of a device of high quality, shedding light on both the HB-related electron delocalization-induced hyper conjugative effects and the many effects found to influence the NH-stretch spectral shift, i.e., the main measurable quantity on which most of the spectroscopic approaches are based.

The aim of this study is complementary to our work which is published in omega and Scientific reports journals^5,8^ to complete our work in this point that we study with the Natural Bond Orbital property to find the mechanism of the stability of the tautomer and rotamers of 2-hydroxyl phenyl azaazulene and -mercapto phenyl azaazulene.

In the current work, we attempt to investigate the electronic structure, stabilities, and bonding characteristics of the 2HPhAZ and 2MPhAZ tautomers and rotamers in gas phase and ethanol at the theoretical level B3LYP/6–311 +  + G(2d,2p). The hybridization of each atom, natural charges (Core, Valence, and Rydberg), the second order perturbation energy (E ^(2)^) of bonding and antibonding orbitals, exact configurations, and Lewis and non-Lewis's electrons have all been considered in the study of the results from natural bonding orbital analysis in gas phase and ethanol.

## Computational details

The gradient-corrected hybrid density functional B3LYP/DFT approach was used for all computations^9,10^. Using this functional^10^ and the 6–311 +  + G (2d, 2p) basis sets^11^ as implemented in the Gaussian 09 program^12^, a comprehensive geometry optimization without symmetry constraints was carried out for each structure. Both Gauss View 5.0.9^13^ and Chem-Craft 1.6^14^ were used to visualize all geometries. The natural bond orbital approach^15^ has also been used to perform the population analysis at the B3LYP/6–311 +  + G (2d, 2p) level of theory using the Gaussian 09 software package. In this process, sets of natural atomic orbitals (NAOs), natural hybrid orbitals (NHOs), and natural boundary orbitals (NBOs) are successively changed from non-orthogonal atomic orbitals (AOs).

Since electron density and other attributes are defined by the fewest number of filled orbitals in the fastest-convergent manner, all these localized basis sets are complete and define the wavefunctions in the most practical manner. The second-order perturbation interaction energy (*E*^*(2)*^), which is defined using the NBO technique, can be used to quantitatively explain this noncovalent bonding-antibonding interaction^16–18^. The estimation of the off-diagonal NBO Fock matrix elements is represented by this energy. From the second-order perturbation method, it may be inferred^15^.

1 $$E^{{({2})}} = \Delta E_{{{\text{ij}}}} = q_{i} (F(i{\text{j}})^{{2}} /\varepsilon_{j} {-}\varepsilon_{i} ),$$

where *q*_*i*_ is the donor orbital occupancy, ε_*i*_ and ε_*j*_ are diagonal elements and (*i*j) is the off-diagonal NBO Fock matrix element.

## Result and discussion

### Geometry optimization

**Figure 2:**
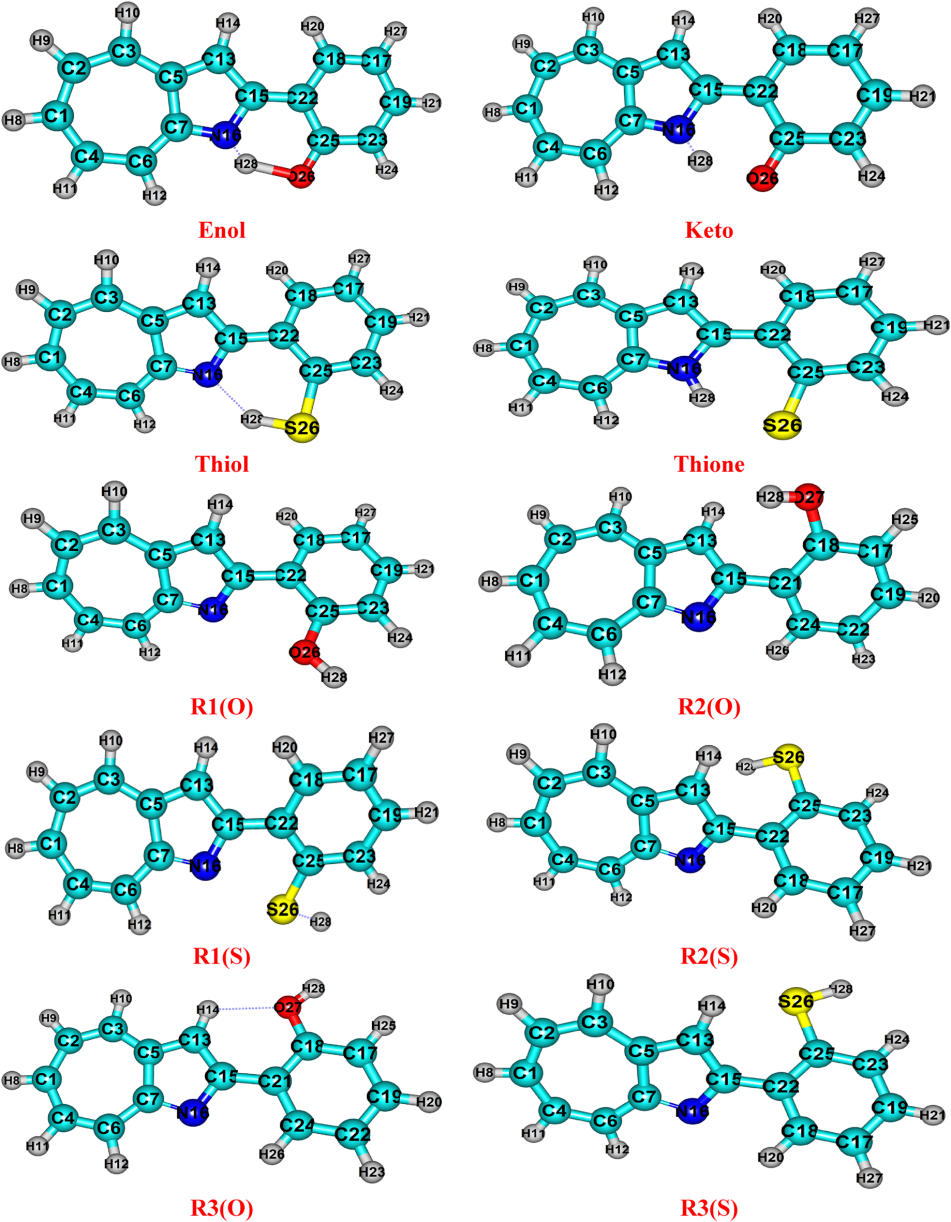
The optimized structural for the tautomers and rotamers of 2HPhAZ and 2MPhAZ at B3LYP/ 6–311 +  + G(2d,2p) level of theory.

Figure 2 lists the tautomeric structures of 2HPhAZ and 2MPhAZ (enolimines/thiol and ketoenamines/thione forms). Table 1 lists all the optimized structural properties determined in gas phase, and ethanol at the B3LYP/6–311 +  + G(2d,2p) level of theory. Table 1 shows that the bond lengths in the tautomeric structure of 2HPhAZ (enolimines and ketoenamines form) are shorter than those in the **thiol** and **thione** forms of the mercapto homologue, but the estimated bond angles have shown the opposite in both gas and ethanol phases. The mercapto analogue's bond angles are less than those of hydroxy structures. Additionally, it has been discovered that the HB length of the N–H–O(S) and the C–N–H bond angle are longer and smaller for the **thiol** and **thione** forms than the **enol** and **keto** forms, respectively. The chemical differences between sulfur and oxygen atoms are to blame for that. The longest hydrogen bond, O(S)…H, is between the **enol** and **thione** forms. The **R3(O)** and **R3(S)** rotamers have the longest O(S)–C bonds among them. The **R2(O)** and **R2(S)** produce the biggest C–O(S)–H bond angle by rotating the phenyl ring. An intriguing feature to note is how the bond length and angle change when the tautomeric structure's σ_F_ values change^19–21^. i.e., σ_F_ is the value of change in factor of the bond length and bond angle when adding any para substituent on the benzene ring; in this case (our compounds), when changed to the tautomeric structures of 2HPhAZ and 2MPhAZ compounds, the value of factor of the bond length and bond angle are changed.

**Table 1:** The selected bond lengths in angstrom (Å) and angles in degrees (◦) calculated for the tautomer and rotamers structures of 2HPhAZ and 2MPhAZ in gas phase, and ethanol using B3LYP/ 6–311 +  + G(2d,2p) level of theory.

Compounds	Bond lengths in Angstrom (Å)						Bond angles in degrees (◦)			
	C-O/S		O/S–H		N–H		< C-O/S–H		< C-N–H	
	Gas	Ethanol	Gas	Ethanol	Gas	Ethanol	Gas	Ethanol	Gas	Ethanol
Enol	1.343	1.356	0.993	1.000	1.703	1.669	107.775	107.329	152.633	152.408
Thiol	1.787	1.784	1.389	1.363	1.725	1.854	93.426	94.804	143.947	141.273
Keto	1.270	1.289	1.630	1.797	1.050	1.027	105.561	105.63	134.229	130.358
Thione	1.760	1.758	2.094	2.110	1.041	1.035	87.650	87.693	129.049	128.643
R1(O)	1.361	1.366	0.962	0.966			109.160	109.259		
R1 (S)	1.797	1.792	1.353	1.346			93.888	94.157		
R2(O)	1.366	1.367	0.964	0.961			109.605	111.33		
R2(S)	1.794	1.789	1.348	1.343			97.887	98.073		
R3(O)	1.369	1.366	0.962	0.966			109.020	109.541		
R3(S)	1.797	1.792	1.349	1.344			95.499	95.446		

### Electronic structure

Enolimines/thiol and ketoenamines/thione are the two π-isoelectronic (tautomeric) structures in which the 2HPhAZ and 2MPhAZ are found. Additionally, research has been done on the three rotameric forms (**R1, R2,** and **R3**). DFT calculations at B3LYP/ 6–311 +  + G(2d,2p) level of theory to determine which form is more stable at the ground state by doing frequency calculations at the same level of calculation, the optimized geometry of the investigated tautomeric structure was verified as a minimum on the potential energy surface. Figure 2 shows the optimized structure for the investigated tautomeric structure.

The C1 symmetry point group is where the optimization structures are found (supporting information). Table 2 displays the total energy and relative energy of the tautomeric and rotameric structures calculated at the B3LYP/6–311 +  + G(2d,2P) in gas and ethanol phases. In case of 2HPhAZ, the studied DFT method qualitatively gives similar tautomer stability orders in the gas phase and ethanol. The ethanol solvent causes some reordering of the relative stability of 2MPhAZ conformers.

**Table 2:** Relative zero-point corrected energies (Δ*E*^0^, kJ/mol), and the energy gap (E_g_), for the tautomer and rotamers structures 2HPhAZ and 2MPhAZ in gas phase, and ethanol at B3LYP/ 6–311 +  + G(2d,2p) level of theory.

Compounds	(Δ*E*^*0*^, kJ/mol)		E_g_(ev)	
	Gas phase	Ethanol	Gas phase	Ethanol
Enol	0.000	0.000	3.098	3.322
Keto	29.492	7.737	2.275	2.617
R1 (O)	54.991	25.992	3.482	3.510
R2(O)	37.731	30.293	3.349	3.458
R3(O)	37.668	37.303	3.399	3.435
Thiol	0.000	19.854	2.980	3.288
Thione	5.254	0.000	1.852	2.299
R1(S)	8.026	24.561	3.189	3.396
R2(S)	10.035	28.004	3.308	3.475
R3(S)	9.178	25.860	3.388	3.510

For tautomerization, the **thiol** form was found to be more stable than the **thione** form in the gas phase. The relative energy difference between **thiol** and **thione** in the gas phase is 5.253 kJ at B3LYP/6–311 +  + G(2d,2P). Stabilization of thiol in the gas phase is expected by the presence of a lower and stronger IHB of **thione**.

On the energy scale, the tautomeric structures are present in a static mixture in the ground state, with the **enol** form being (29.492 and 7.737 kJ) more stable than the **keto** form in gas phase and ethanol, respectively (Table 2). It has been found that **keto/thione** form has the lowest HOMO–LUMO gap (2.275/1.852 eV) followed by **enol/thiol** forms (3.098/2.980 eV).

In the ground state of rotamers in statical mixes, the **R3(O)** structure is more stable than the **R2(O)** and **R1(O)** structures, but the **R1(S)** structure is more stable than the **R3(S)** and **R2(S)** structures (Table 2). The stability of **R2(O)** and **R3(O)** can be attributed to the possible intramolecular H bonding interaction of the C–H…N as shown in Fig. 2. The slightly higher stability of **R3(S)** than **R2(S)** can be due to the anti-position present between the S26-H28 group and the nitrogen of the azaazulene ring, given the possibility of H28 interaction with the sulfur atom. The highest gap is in the rotamers. It is widely known that a molecule's reactivity increases with decreasing energy gaps^22,23^. As a result, it is anticipated that the **keto/thione** form will be more chemically reactive than **enol/thiol** and their rotamer. The **keto/thione** compound can function as both an electron acceptor and donor. The results of the MO calculation show that the computed reactivity in the gas phase and ethanol of the studied compounds increases in the order.

For 2HPhAZ, **enol > keto > R3(O) > R2(O) > R1(O)** in gas phase but in ethanol the order of stability is **Enol > keto > R1(O) > R2(O) > R3(O).**

In case of 2MPhAZ, **thiol > thione > R1(S) > R3(S) > R2(S)** in gas phase. In ethanol, the order of stability becomes **thione > thiol > R1(S) > R3(S) > R2(S)** in 2MPhAZ. Our results are agreeing with the previous results that of 2-(2-Hydroxyphenyl)-1-aza azulene and 2-(2-Mercaptophenyl)-1-aza azulene^5,8^.

### Character of natural hybrid orbital (NHO) on the tautomeric structure

A natural atomic orbital (NAO) is a valence-shell atomic orbital that was created by diagonalizing the localized block of a given molecule's full density matrix, is connected to the atom in question via basis functions *X*_i_ (A), and simultaneously satisfies the orthonormality and maximum occupancy conditions. While NAOs in a single atom simply coincide with natural orbitals, in polyatomic molecules they largely maintain their one-center nature (as opposed to natural orbitals, which get delocalized over all nuclear centers). Since a single center in polyatomic molecules is surrounded by a molecular electron density, NAOs is regarded as offering the best explanation of this position. Additionally, an NHO is produced via a unitary transformation of a directed hybrid orbital that is symmetrically orthogonalized and centered on a specific atom. Finally, an NBO is an orbital produced from NHOs in the simple bond orbital diagram. Thus, for a localized σ-bond between atoms A and B, the NBO is defined as:

2 $$\sigma_{{{\text{AB}}}} = C_{{\text{A}}} h_{{\text{A}}} + C_{{\text{B}}} h_{{\text{B}}}$$

where *h*_A_ and *h*_B_ are the natural hybrids centered on atoms A and B. Therefore, the NBOs thoroughly agree to the representation of localized bonds and lone pairs as basic units of molecular structure, and hence it will be probable to handily take ab initio wavefunctions in terms of the classical Lewis structure concepts by converting these functions to the NBO forms^24^. In Table 3, we produce the resulting natural atomic hybrids *h*_A_ on some atoms with the polarization coefficient *C*_A_ for each hybrid (in explanations) in the equivalent NBO. The assessment of the results described in Table 3 in gas phase, and ethanol shows that:

**Table 3:** Calculated natural hybrid (NHOs) and the polarization coefficient for each hybrid in the equivalent NBO (in explanations) for the studied the tautomers and rotamers structures of 2HPhAZ and 2MPhAZ in gas phase, and ethanol at B3LYP/6–311 +  + G(2d,2p) level of theory.

Compounds	Bond			C25-O26/ S26				C15-N16	
	Hybrids	O26/ S26		C25		C15		N16	
		Gas phase	Ethanol	Gas phase	Ethanol	Gas phase	Ethanol	Gas phase	Ethanol
Enol	B3LYP	sp^1.79^d^0.01^(0.8142)	sp^1.82^d^0.01^(0.8159)	sp^2.90^d^0.00^(0.5806)	sp^2.98^d^0.00^(0.5782)	sp^2.54^d^0.00^(0.6379)	sp^2.55^d^0.00^(0.6371)	sp^1.97^d^0.01^(0.7702)	sp^1.97^d^0.01^(0.7708)
Thiol	B3LYP	sp^4.55^d^0.02^(0.6478)	sp^4.69^d^0.08^(0.6757)	sp^2.62^d^0.02^(0.7635)	sp^2.96^d^0.01^(0.7372)	sp^2.88^d^0.00^(0.6165)	sp^2.26^d^0.00^(0.6325)	sp^2.05^d^0.00^(0.7870)	sp^1.64^d^0.00^(0.7746)
Keto	B3LYP	sp^1.00^d^0.00^(0.8640)	sp^1.61^d^0.01^(0.8036)	sp^1.00^d^0.00^(0.5034)	sp^2.43^d^0.00^(0.5952)	sp^2.94^d^0.00^(0.6164)	sp^2.94^d^0.00^(0.6168)	sp^2.11^d^0.00^(0.7874)	sp^1.50^d^0.00^(0.7871)
Thione	B3LYP	sp^4.78^d^0.02^(0.6491)	sp^4.56^d^0.02^(0.6535)	sp^2.42^d^0.02^(0.7607)	sp^2.45^d^0.02^(0.7569)	sp^2.82^d^0.00^(0.6156)	sp^2.85^d^0.00^(0.6173)	sp^2.00^d^0.00^(0.7880)	sp^1.50^d^0.00^(0.7867)
R1 (O)	B3LYP	sp^1.87^d^0.01^(0.8177)	sp^1.82^d^0.01^(0.8184)	sp^3.05^d^0.00^(0.5757)	sp^3.06^d^0.00^(0.5747)	sp^2.36^d^0.00^(0.6454)	sp^2.37^d^0.00^(0.6428)	sp^1.97^d^0.01^(0.7639)	sp^1.93^d^0.01^(0.7660)
R1 (S)	B3LYP	sp^4.60^d^0.06^(0.6765)	sp^4.46^d^0.06^(0.6800)	sp^2.97^d^0.01^(0.7365)	sp^3.01^d^0.01^(0.7332)	sp^2.42^d^0.00^(0.6439)	sp^2.42^d^0.00^(0.6426)	sp^1.98^d^0.01^(0.7651)	sp^1.93^d^0.01^(0.7662)
R2 (O)	B3LYP	sp^3.67^d^0.02^(0.8711)	sp^3.65^d^0.02^(0.8721)	sp^2.25^d^0.01^(0.5784)	sp^2.23^d^0.01^(0.5774)	sp^2.64^d^0.00^(0.6510)	sp^2.47^d^0.00^(0.6436)	sp^2.03^d^0.01^(0.7570)	sp^1.99^d^0.01^(0.7654)
R2 (S)	B3LYP	sp^4.55^d^0.02^(0.6809)	sp^4.46^d^0.02^(0.6811)	sp^3.07^d^0.02^(0.7324)	sp^3.06^d^0.02^(0.7322)	sp^2.44^d^0.00^(0.6442)	sp^2.43^d^0.00^(0.6427)	sp^2.01^d^0.01^(0.7648)	sp^1.94^d^0.01^(0.7661)
R3(O)	B3LYP	sp^3.76^d^0.02^(0.8601)	sp^3.74^d^0.02^(0.8611)	sp^2.35^d^0.00^(0.5775)	sp^2.32^d^0.00^(0.5769)	sp^2.46^d^0.00^(0.6439)	sp^2.48^d^0.00^(0.6427)	sp^2.01^d^0.01^(0.7651)	sp^1.98^d^0.01^(0.7661)
R3(S)	B3LYP	sp^4.51^d^0.02^(0.6825)	sp^4.51^d^0.02^(0.6825)	sp^3.08^d^0.02^(0.7309)	sp^3.08^d^0.02^(0.7309)	sp^2.44^d^0.00^(0.6448)	sp^1.00^d^0.00^(0.7991)	sp^2.03^d^0.01^(0.7644)	sp^1.00^d^0.00^(0.6011)

The p characteristics of sulfur NHO σ_C25-S26_ and nitrogen NHO σ_C15-N16_ bond orbitals increase with increasing Hammett constant in gas phase, and ethanol due to stretching of the C25-S26 bond of the thiol and thione tautomer. The C25-O26 bond of enolimines and ketoenamines tautomers shortens, in gas phase, and ethanol causing orbitals to contract with increasing Hammett constant, in contrast to the p characteristics of oxygen NHO σ_C25-O26_ and nitrogen NHO σ_C15-N16_. While the predicted d contributions of S atoms, which are insignificant and nearly equal, determine that 3d orbitals are not important for the bonding of the thiol and thione tautomer in gas phase, and ethanol.

The O(S)-C sigma bond's interaction becomes weaker. These compounds exhibit strong delocalized *n*_*O(S)*_ **—> **σ*_C25-O26(S26)_ and *n*_*O(S)*_ **—> **σ*_C15-N16_ as well as significant hyper conjugative interaction, which results in partial p character (Table 4). Additionally, Table 4 shows that *E*^(2)^ rises in gas phase, and ethanol when the investigated compounds' Hammett constants rise, extending the (O)S-C bond as a result. Table 2's stability order reveals that the **enol/thiol** structure has higher Relative zero-point corrected energies (Δ*E*^0^, kJ/mol), values than the **keto/thione** structure in gas phase. The **enol** and **thiol** structure being more stable than the **keto** and **thione** structures in gas phase. The ethanol solvent causes some reordering of the relative stability of 2HPhAZ and 2MPhAZ conformers. The relative energy difference between **thiol** and **thione** in the gas phase is 5.253 kJ at B3LYP/6–311 +  + G (2d,2P). For rotamer, **R3(O)** structure have higher stability than **R2(O)** and **R1(O)** structures, while **R1(S)** structure has higher stability than **R3(S)** and **R2(S)** structures (Table 2).

**Table 4:** Second order delocalization energies (*E*^(2)^) for the studied compounds in gas phase, and ethanol at B3LYP/6–311 +  + G(2d,2p) level of theory, all values are in kcal/mol.

Compounds	n_O26/S26_ — > σ*_C25-O26/S26_		n_O26/S26_ — > σ*_C15-N16_	
	Gas phase	Ethanol	Gas phase	Ethanol
Enol	43.33	42.85	21.44	20.65
Keto	36.44	36.01	28.70	26.98
R1(O)	74.43	73.55	42.71	42.11
R2(O)	65.65	65.11	57.21	56.88
R3(O)	81.25	80.66	64.25	63.54
Thiol	44.35	43.31	50.62	51.01
Thione	56.31	56.10	42.42	40.98
R1(S)	66.87	65.77	64.11	65.02
R2(S)	78.88	77.54	75.69	77.10
R3(S)	80.48	82.09	82.82	80.11

### Donor–acceptor (bond–anti-bond) interactions

Each valence bonding NBO (σ_AB_) in the NBO analysis^24^ must be coupled with a corresponding valence antibonding NBO (σ*_AB_), to compute the span of the valence space:

3 $$\sigma *_{{{\text{AB}}}} = C_{{\text{A}}} h_{{\text{A}}} - C_{{\text{B}}} h_{{\text{B}}}$$

For example, the Lewis σ-type (donor) NBOs are matched by the non-Lewis σ*-type (acceptor) NBOs that are properly vacant in an ideal Lewis structure picture. The universal transformation to NBOs leaves vacant orbitals in the formal Lewis structure. As a result, the filled NBOs of the natural Lewis's structure can accurately reflect covalency effects in molecules. Given that the non-covalent delocalization effects are linked to σ **— > **σ* interactions between filled (donor) and empty (acceptor) orbitals, it is only logical to refer to them as being of the donor–acceptor, charge transfer, or generalized "Lewis's base-acid" Lewis's type. The anti-bonds reflect unutilized valence-shell capacity and span areas of the atomic valence spaces that are formally unsaturated by covalent bond formation. Weak occupancies of the valence anti-bonds represent actual "delocalization effects," or irreducible deviations from the idealized localized Lewis picture. To account for the donor–acceptor (bond-anti-bonds) interactions, all potential interactions between "filled" (donor) Lewis-type NBOs and "empty" (acceptor) non-Lewis NBOs are examined in the NBO analysis. The energies of these interactions are then estimated using second-order perturbation theory. These interactions (or energy stabilization) are referred to as "delocalization" corrections for the zeroth-order natural Lewis structure.

Table 5 reports the most significant interaction between "unoccupied" (acceptor) non-Lewis NBOs and "occupied" (donor) Lewis-type NBOs. The lp (S or O) participates as a donor and the BD*(O(S)-C) anti-bond as an acceptor [lp (S or O) **— > **BD*(O(S)-C)], with charge transfer energy values at the B3LYP level. These results of the NBO analysis are collected in Table 5 in gas phase, and ethanol. This shows that increasing the Hammett constants of these studied compounds leads to a decrease in charge transfer energy. It may be concluded that the strength of the O(S)-C bond only minimally changes in the studied compounds because the amount of destabilization energy predicted using the NBO technique does not significantly alter in each of the compounds, which is consistent with our calculated results^5^ and the experimental results^25^.

**Table 5:** The second-order perturbation energies *E*^(2)^ (kcal/mol) corresponding to the most important charge transfer interactions (donor **— > **acceptor) in the studied compounds in gas phase, and ethanol by using B3LYP/6–311 +  + G(2d,2p) method.

Don. NBO	Acc NBO	*E*^(2)^ (kcal/mol)									
		Enol	Thiol	Keto	Thione	R1(O)	R1(S)	R2(O)	R2(S)	R3(O)	R3(S)
lp(O/S) in gas	BD*(O/S -C26) in gas	23.33	26.87	18.54	29.32	45.90	41.25	25.14	25.69	31.25	32.12
lp(O/S) in ethanol	BD*(O/S -C26) in ethanol	22.43	25.85	19.01	28.12	44.66	40.88	24.33	25.03	30.44	33.03
lp(N) in gas	BD*(N-C15) in gas	14.24	17.21	12.08	22.10	12.21	16.32	18.22	28.88	19.78	23.65
lp(N) in ethanol	BD*(N-C15) in ethanol	14.00	18.21	11.88	21.86	11.57	15.78	17.54	26.77	19.20	16.69

lp is the lone pair NBO in the plane.

The calculated natural orbital occupancy, repeatedly known as the orbital's "natural population," is shown in Table 6 in gas phase, and ethanol. It is observed that the maximum occupancy for σ _C-O(S)_ and σ* _C-O(S)_ bond orbitals is obtained for **R1(O)** and **R3(S)** 1.99338 (1.99348), 0.02224 (0.02295) in gas phase and in ethanol, respectively and 1.98245 (1.98302), 0.02668 (0.02817), in gas phase, and, in ethanol, respectively. As we explained above, small occupancies of the antibound orbitals relate, in Hartree Fock theory, to a complicated change from the idealized Lewis picture and thus to small non-covalent corrections to the picture of localized covalent bonds. The resulting p character of the corresponding sulfur natural hybrid orbital (NHO) of the σ_C-O(S)_ bond orbital is also related to the impact on molecular stability and geometry (bond lengths) of decreased occupancy of the localized σ_C-O(S)_ orbital in the idealized Lewis structure or increased occupancy of σ*_C-O(S)_ of the non-Lewis orbital. In Table 3, for selected compounds at B3LYP level [**thiol**, **thione**, **R1(S)**, **R2(S)**, and **R3(S)**], the p characters of sulfur σ _C-S_ values are 4.55(4.69), 4.78(4.56), 4.60(4.46), 4.55(4.46), and 4.51(4.51), in gas phase, and ethanol, respectively. Like this, the lengths of the C-S bonds are 1.787(1.784), 1.760(1.758), 1.797(1.792), 1.794(1.789), and 1.797(1.792), in gas phase, and ethanol, respectively. For [**enol**, **keto**, **R1(O)**, **R2(O)** and **R3(O)**], the p characters of oxygen σ _C-O_ values are 1.79(1.82), 1.00(1.61), 1.87(1.82), 3.67(3.65), and 3.76(3.74), in gas phase, and ethanol, respectively. Like this, the lengths of the C-O bonds are 1.343(1.356), 1.270(1.289), 1.361(1.366), 1.366(1.367), and 1.369(1.366), in gas phase, and ethanol, respectively. Therefore, the results indicate that the C-O(S) bond lengths of these compounds are basically controlled by the p character of these hybrid orbitals and by the nature of the C-O(S) bond.

**Table 6:** The important calculated valence non-Lewis and Rydberg non-Lewis, σ _C-O(S)_ and σ*_C-O(S)_ bond orbital occupancies in gas phase, and ethanol at B3LYP/6–311 +  + G(2d,2p).

	Enol	Thiol	Keto	Thione	R1(O)	R1(S)	R2(O)	R2(S)	R3(O)	R3(S)
σ_C25-O/S26_ in gas phase	1.99236	1.97809	1.99291	1.98009	1.99338	1.98159	1.97658	1.98230	1.98704	1.98245
σ_C25-O/S26_ in ethanol	1.99241	1.98202	1.99247	1.98006	1.99348	1.98206	197,628	1.98257	1.97659	1.98302
σ_C15-N16_ in gas phase	1.98071	1.97429	1.98085	1.98219	1.97955	1.98000	1.98895	1.97879	1.97996	1.97893
σ_C15-N16_ in ethanol	1.98092	1.95645	1.97556	1.97645	1.97990	1.98011	1.97972	1.97911	1.98030	1.97910
lp(O/S) in gas phase	1.97378	1.98458	1.96699	1.98348	1.97955	1.98941	1.96785	1.98699	1.97924	1.98825
lp(O/S) in ethanol	1.97559	1.98477	1.97239	1.98229	1.97969	1.98964	1.97839	1.98710	1.97850	1.98846
lp(N) in gas phase	1.88629	1.89328	1.89739	1.91608	1.93333	1.91908	1.91323	1.93496	1.93344	1.93493
lp(N) in ethanol	1.87803	1.89690	1.83173	1.91178	1.93738	1.92479	1.93603	1.93791	1.93724	1.93828
σ*_C25-O/S26_ in gas phase	0.02026	0.02328	0.01265	0.01238	0.02224	0.02582	0.01524	0.02530	0.01434	0.02668
σ*_C25-O/S26_ in ethanol	0.02209	0.02368	0.12318	0.01699	0.02295	0.02721	0.01487	0.02593	0.01473	0.02817
σ*_C15-N16_ in gas phase	0.54345	0.51578	0.02309	0.02572	0.49912	0.50768	0.01715	0.50287	0.01613	0.51806
σ*_C15-N16_ in ethanol	0.54488	0.51689	0.02487	0.02351	0.49649	0.50578	0.53287	0.50437	0.01854	0.51400
Valence non-Lewis in gas phase	1.99973	1.99978	1.99975	1.99921	1.99974	1.99989	1.99972	1.99979	1.99975	1.99989
Valence non-Lewis in ethanol	1.99974	1.99987	1.99978	1.99954	1.99972	1.99988	1.99975	1.99989	1.99975	1.99989
Rydberg non-Lewis in gas phase	0.00278	0.00264	0.00292	0.00346	0.00290	0.00438	0.00265	0.00479	0.00235	0.00476
Rydberg non-Lewis in ethanol	0.00246	0.00224	0.00289	0.00364	0.00229	0.00483	0.00266	0.00492	0.00255	0.00476

lp is the lone pair NBO in the plane.

### Natural population analysis

The natural population analysis (NPA) was evaluated using natural atomic orbital occupancies^26^. Table 7 in gas phase, and ethanol displays the molecular charge distribution on the skeletal atoms of the rotomeric and tautomeric forms of 2HPhAZ and 2MPhAZ. In general, it is seen that the strong negative and positive partial charges on the skeleton atoms—particularly O, N, and S—increase as the examined compounds' Hammett constants increase. The most electronegative center charges of [**enol**, **keto**, **R1(O)**, **R2(O)**, and **R3(O)**] are −0.58236(0.60716), −0.53433(−0.52220), −0.48994(−0.55563), −0.5088(−0.56684), −0.5210(−0.57059), and −0.69700(−0.74332), −0.71822(−0.83839), −0.65636 (−0.69924), −0.6867(−0.70641), and −0.6831(−0.70657), in gas phase, and ethanol respectively, which are accumulated on N and O atoms. While [**thiol**, **thione**, **R1(S)**, **R2(S)**, and **R3(S)**], the most electronegative center charges on N and S atoms are −0.60289(−0.56601), −0.5412(−0.53769), −0.5132(−0.55665), −0.5113(−0.56395), −0.5129(−0.56467), and −0.02443(−0.01982), −0.27762(−0.43861), −0.10941(−0.05444), −0.04712(−0.02862), and 0.0578(−0.02977), in gas phase, and ethanol respectively. According to the electrostatic point of view of the molecule, these electronegative atoms tend to donate electrons. Also, it is found that the most electropositive center charges of [**enol**, **keto**, **R1(O)**, **R2(O)**, and **R3(O)**] and [**thiol**, **thione**, **R1(S)**, **R2(S)**, and **R3(S)**] are accumulated on C7, C15, and C25 atoms (cf. Table 7). These electropositive atoms have a propensity to accept electrons from the perspective of the molecule's electrostatics. Table 7 lists each electronegative and electropositive atom's native electronic configuration. According to the distribution of partial charges on the skeleton atoms, electrostatic attraction or repulsion between atoms can contribute significantly to intra- and intermolecular interaction.

**Table 7:** Atomic charge distribution described in terms of natural population analysis (NPA) for the studied compounds in gas phase, and ethanol by using B3LYP/6–311 +  + G(2d,2p) method.

	Enol	Thiol	Keto	Thione	R1(O)	R1(S)	R2(O)	R2(S)	R3(O)	R3(S)
C1 in gas phase	-0.16588	–0.16422	–0.19673	–0.5690	–0.17080	–0.1695	–0.1613	-0.1584	–0.1727	–0.1672
C1 in ethanol	–0.19941	–0.20172	–0.16537	–0.18866	–0.16690	–0.15447	–0.16307	–0.15853	–0.16709	–0.15176
C2 in gas phase	–0.20506	–0.20645	–0.18806	–0.2014	–0.21512	–0.2138	–0.2071	–0.2127	–0.2142	–0.2138
C2 in ethanol	–0.19959	–0.13607	–0.18607	–0.18011	–0.21285	–0.21304	–0.20808	–0.21026	–0.21256	–0.21107
C3 in gas phase	–0.11955	–0.13963	–0.13884	–0.1349	–0.12243	–0.1215	–0.1234	–0.1125	–0.1236	–0.1120
C3 in ethanol	–0.13183	–0.03984	–0.11380	–0.12132	–0.12298	–0.11808	–0.12148	–0.11567	–0.12317	–0.10876
C4 in gas phase	–0.20004	–0.20313	–0.15073	0.73914	–0.18809	–0.1854	–0.1971	–0.2021	–0.1878	–0.1881
C4 in ethanol	–0.10506	–0.20032	–0.15604	–0.07596	–0.18850	–0.21053	–0.19219	–0.19394	–0.19564	–0.20975
C5 in gas phase	–0.06764	–0.25527	–0.04805	–0.0659	–0.07527	–0.0733	–0.0672	–0.0733	–0.0738	–0.0714
C5 in ethanol	–0.06638	–0.12134	–0.05409	–0.05101	–0.08120	–0.07957	–0.07255	–0.07818	–0.07910	–0.07723
C6 in gas phase	–0.14915	–0.14289	–0.20378	–0.2764	–0.14398	–0.1442	–0.1370	–0.1341	–0.1481	–0.1418
C6 in ethanol	–0.19480	–0.14964	–0.16575	–0.18868	–0.15308	–0.14190	–0.14926	–0.14494	–0.15422	–0.13930
C7 in gas phase	0.21167	0.18890	0.22981	0.21355	0.18714	0.19214	0.19170	0.18670	0.19207	0.18711
C7 in ethanol	0.20011	0.16813	0.21993	0.21802	0.18056	0.18024	–0.18364	0.17780	0.18246	0.17875
C13 in gas phase	–0.25869	–0.87975	–0.24933	–0.2413	–0.26482	–0.2618	–0.3094	–0.2803	–0.2543	–0.2655
C13 in ethanol	–0.27109	–0.31545	–0.25853	–0.25116	–0.27539	–0.27226	–0.28988	–0.28425	–0.27459	–0.27778
C15 in gas phase	0.22868	0.05725	0.25384	0.25612	0.21258	0.21168	0.21496	0.20875	0.20507	0.20507
C15 n ethanol	0.20785	0.32704	0.24851	0.24771	0.19985	0.19580	–0.19179	0.19274	0.19051	0.19088
N16 in gas phase	–0.58236	–0.60289	–0.53433	–0.5412	–0.48994	–0.5132	–0.5088	–0.5113	–0.5210	–0.5129
N16 in ethanol	–0.60716	–0.56601	–0.52220	–0.53769	–0.55563	–0.55665	–0.56684	–0.56395	–0.57059	–0.56467
C17 in gas phase	–0.24695	–0.24303	–0.26237	–0.2422	–0.23959	–0.2247	–0.2528	–0.2211	–0.2811	–0.2214
C17 in ethanol	–0.25566	–0.22810	–0.28993	–0.25793	–0.25197	–0.22974	–0.26533	–0.26649	–0.28099	–0.22884
C18 in gas phase	–0.13887	–0.61862	–0.14385	–0.1462	–0.14321	–0.1461	0.35002	–0.1338	0.34961	–0.1324
C18 in ethanol	–0.15229	–0.16652	–0.15189	–0.15134	–0.15619	–0.15793	–0.34626	–0.06527	0.34849	–0.15498
C19 in gas phase	–0.17230	–0.17539	–0.16098	–0.1697	–0.17461	–0.1778	–0.1663	–0.1776	–0.1708	–0.1782
C19 in ethanol	–0.18804	–0.18657	–0.17880	–0.18282	–0.18591	–0.18805	0.18157	–0.18931	–0.18199	–0.23480
C22 in gas phase	–0.16354	0.72442	–0.19917	–0.1527	–0.12282	–0.1101	–0.1623	–0.1105	–0.1229	–0.1077
C22 in ethanol	–0.15962	–0.16897	–0.20667	–0.16102	–0.13693	–0.11513	–0.14816	–0.14456	–0.13562	–0.11493
C23 in gas phase	–0.23910	–0.23362	–0.26583	–0.22041	–0.27668	–0.2269	–0.2548	–0.2299	–0.2335	–0.2325
C23 in ethanol	–0.25555	–0.23438	–0.29957	–0.24746	–0.27993	–0.23643	–0.24481	–0.23739	–0.24919	–0.12436
C25 in gas phase	0.37617	–0.48595	0.43933	–0.12756	0.35734	–0.1174	–0.5649	–0.1310	–0.1398	–0.1270
C25 in ethanol	0.35354	–0.14899	0.40459	–0.13609	–0.34650	–0.13237	–0.16298	–0.14337	–0.16363	–0.18300
O26/S26 in gas phase	–0.69700	0.02443	–0.71822	–0.27762	–0.65636	0.10941	–0.6867	0.04712	–0.6831	0.0578
O26/S26 in ethanol	–0.74332	–0.01982	–0.83839	–0.43861	–0.69924	0.05444	–0.70641	0.02862	–0.70657	0.02977

## Conclusion

Using the DFT/B3LYP/6–311 +  + G (2d, 2p) level of theory, the electronic for tautomeric and rotameric structures of 2HPhAZ and 2MPhAZ analogues is explored in gas phase, and ethanol. **Enol/thiol** is more stable than **keto/thione** form for tautomer in the statically mixed ground state in gas phase, which is considered in all computations in this article. The ethanol solvent causes some reordering of the relative stability of 2HPhAZ and 2MPhAZ conformers. For rotamer, **R3(O)** structure have higher stability than **R2(O)** and **R1(O)** structures, while **R1(S)** structure has higher stability than **R3(S)** and **R2(S)** structures. According to our findings, an intriguing aspect to note is how the bond length and angle alter as a function of the tautomeric structure's σ_F_ values. The examined compounds' increasing Hammett constants result in a rise in the p characteristics of the sulfur NHO σ _C-S(O)_ bond orbitals. There is a strong hyperconjugated interaction between *n*_*O*(S)_**—> **σ*_C25-O26(S26)_ and *n*_*O*(S)_ **—> **σ*_C15-N16_ in the compounds studied. The weakness in the O(*S*)-C sigma bond is due to *n*_*O(S)*_— > σ*_C25-O26(S26)_ and *n*_*O(S)*_**—> **σ*_C15-N16_ delocalization, which is responsible for the longer O(*S*)-C bond lengths and hence contributes to the tautomerization of 2HPhAZ and 2MPhAZ. With an increase in the examined compounds' Hammett constants, the charge transfer energy reduces. The intra- and intermolecular interactions can be significantly influenced by the electrostatic attraction or repulsion between atoms. The NBO analysis focuses on the availability of a device of high quality, shedding light on both the HB-related electron delocalization-induced hyperconjugate effects and the many effects found to influence the NH-stretch spectral shift, i.e., the main measurable quantity on which most of the spectroscopic approaches are based.

### Supplementary Information

Supplementary Information.

## Data Availability

All data generated or analyzed during this study are included in this published article [and its supplementary information files].

## References

[1] Abe N, Gunji T (2010). The chemistry of Azaazulenes. Heterocycles.

[2] Kimura M (1981). The chemistry of Aza-Azulenes. J. Synth. Org. Chem. Jpn..

[3] Turányi T, Zalotai L, Dóbé S, Bérces T (2002). Effect of the uncertainty of kinetic and thermodynamic data on methane flame simulation results. Phys. Chem. Chem. Phys..

[4] Oda, M., Ayumi, S., Rie, T., Yurie, F., Ryuta, M., Abe, T., & Kuroda S. Synthesis, Molecular Structure, and Properties of 2-(2-Hydroxyphenyl)-1-azaazulene. *Eur. J. Org. Chem.* 2231–2236 (2012).

[5] El-Meligy AB, El-Demerdash SH, Abdel-Rahman MA, Mahmoud AM, Taketsugu T, El-Nahas AM (2022). Structures, energetics, and spectra of (NH) and (OH) tautomers of 2-(2-Hydroxyphenyl)-1-azaazulene: a density functional theory/time-dependent density functional theory study. ACS Omega.

[6] Foster JP, Weinhold F (1980). Natural hybrid orbitals. J. Am. Chem. Soc..

[7] Reed AE, Curtiss LA, Weinhold F (1988). Intermolecular interactions from a natural bond orbital, donor-acceptor viewpoint. Chem. Rev..

[8] El-Demerdash Safnaz H, Halim Shimaa Abdel, El-Nahas Ahmed M, El-Meligy Asmaa B (2023). A density functional theory study of the molecular structure, reactivity, and spectroscopic properties of 2-(2-mercaptophe nyl)-1-azaazulene tautomers and rotamers. Sci. Rep..

[9] Becke AD (1993). A new mixing of Hartree-Fock and local density-functional theories. J. Chem. Phys..

[10] Becke AD (1993). Densityfunctional thermochemistry, III: The role of exact exchange. J. Chem. Phys..

[11] Lee C, Yang W, Parr RG (1988). Development of the Colle-Salvetti correlation-energy formula into a functional of thse0e electron density. Phys. Rev. B Condens. Matter..

[12] Stefanov, B., Liu, B. G., Liashenko, A., Piskorz, P., Komaromi, I., Martin, R. L., Fox, D. J., Keith, T., Al-Laham, M. A., Peng, C. Y., Nanayakkara, A., Challacombe, M., Gill, P. M. W., Johnson, B., Chen, W., Wong, M. W., Gonzalez, C., Pople, J. A., Gaussian, Inc., Pittsburgh PA. (2003).

[13] Frisch, M., Trucks, J. G. W., Schlegel, H. B., Scuseria, G. E., *et al.* Gaussian, Inc., Wallingford CT, (2009).

[14] GaussView, Version 5, Dennington, R.; Keith, T.; Millam, J. Semichem Inc., Shawnee Mission KS, (2009).

[15] http://www.chemcraftprog.com.

[16] Chocholoušová J, Špirko V, Hobza P (2004). first local minimum of the formic acid dimer exhibits simultaneously red-shifted O-H…O and improper, blue-shifted C–H…O hydrogen bonds. Phys. Chem. Chem. Phys..

[17] Reed AE, Weinhold F (1985). Natural localized molecular orbitals. J. Chem. Phys..

[18] Reed AE, Weinstock RB, Weinhold F (1985). Natural population analysis. J. Chem. Phys..

[19] Reed AE, Weinhold F (1983). Natural bond orbital analysis of near-Hartree–Fock water dimer. J. Chem. Phys..

[20] Xiao-Hong Li, Zheng-Xin T, Xian-Zhou Z (2009). Natural bond orbital (NBO) population analysis of para-substituted S-Nitroso-thiophenols. J. Mol. Struct.: Theochem..

[21] Pross A, Radom L, Taft RW (1980). Theoretical approach to substituent effects Phenols and phenoxide ions. J. Org. Chem..

[22] Wang L, Cao C, Cao C (2019). Substituent effects on the stretching vibration of C═N in multi-substituted benzylideneanilines. J. Phys. Org. Chem..

[23] Cai T, Xu L, Anderson MR, Ge Z, Zuo T, Wang X, Olmstead MM, Balch AL, Gibson HW, Dorn HC (2006). Structure and enhanced reactivity rates of the D5h Sc3N@C80 and Lu3N@C80 metallofullerene isomers: the importance of the Pyracylene Motif. J. Am. Chem. Soc..

[24] Chermette H (1999). Chemical reactivity indexes in density functional theory. J. Comput. Chem..

[25] Reed AE, Curtiss LA, Weinhold F (1988). Intermolecular interactions from a natural bond orbital, donor-acceptor viewpoint. Chem. Rev..

[26] Luo YR (2003). Handbook of Bond Dissociation Energies in Organic Compounds.

