# Supplementary material for: DFT study, and natural bond orbital (NBO) population analysis of 2-(2-Hydroxyphenyl)-1-azaazulene tautomers and their mercapto analouges
Source: Sci Rep. 2024 Jan 2;14:219. doi: 10.1038/s41598-023-50660-w (PMC10762136; doi:10.1038/s41598-023-50660-w)
Supplement: Supplementary file 1 — Supplementary Information. [file 41598_2023_50660_MOESM1_ESM.docx]

**DFT Study, and Natural Bond Orbital (NBO) Population Analysis of 2-(2-Hydroxyphenyl)-1-azaazulene Tautomers and their Mercapto Analouges**

**Shimaa Abdel Halim^a,^ *, Safinaz H. El-Demerdash^b^, Ahmed M. El-Nahas,^b^ Asmaa B. El-Meligy,^b,^***

*^a^Chemistry Department, Faculty of Education, Ain Shams University, Cairo, 1171, Egypt*

*^b^Chemistry Department, Faculty of Science, Menoufia University, Shebin El-Kom 32512, Egypt*

****Corresponding Author***

*E-mail*: [shimaabdelhalim@edu.asu.edu.eg](mailto:shimaabdelhalim@edu.asu.edu.eg) (Shimaa Abdel Halim)

[asmaaphys@yahoo.com](mailto:asmaaphys@yahoo.com) (Asmaa B. El-Meligy)


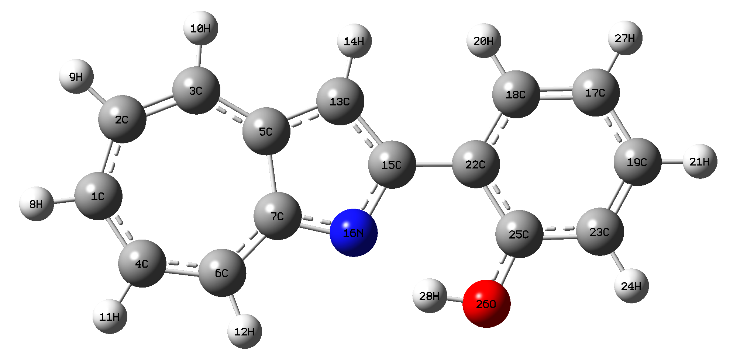


optimized geometries of **enol**

6 -4.684439000 0.160212000 -0.000018000

6 -4.227374000 -1.164181000 0.000023000

6 -2.927927000 -1.638858000 0.000043000

6 -3.960148000 1.343963000 -0.000048000

6 -1.734476000 -0.907359000 0.000028000

6 -2.577313000 1.531437000 -0.000047000

6 -1.584478000 0.563778000 -0.000014000

1 -5.761449000 0.275421000 -0.000026000

1 -5.001912000 -1.921037000 0.000041000

1 -2.815461000 -2.717231000 0.000075000

1 -4.550486000 2.251760000 -0.000078000

1 -2.218769000 2.553384000 -0.000076000

6 -0.435236000 -1.403578000 0.000049000

1 -0.157575000 -2.443258000 0.000079000

6 0.429767000 -0.288248000 0.000022000

7 -0.275676000 0.879554000 -0.000020000

6 4.002005000 -1.472684000 -0.000005000

6 2.621282000 -1.466884000 0.000019000

6 4.690506000 -0.255318000 -0.000035000

1 2.089483000 -2.407717000 0.000036000

1 5.772164000 -0.246169000 -0.000056000

6 1.883369000 -0.269169000 0.000017000

6 3.999472000 0.941762000 -0.000033000

1 4.516696000 1.890577000 -0.000050000

6 2.599935000 0.956780000 -0.000002000

8 1.991632000 2.153731000 0.000017000

1 4.543063000 -2.407913000 -0.000006000

1 1.011241000 1.995546000 0.000073000


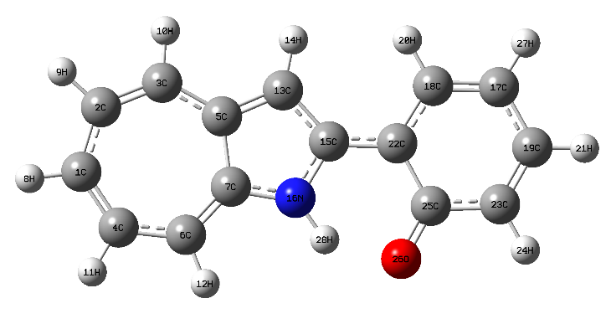


optimized geometries of **keto**

6 4.674513000 0.189530000 0.000084000

6 4.219320000 -1.147434000 0.000125000

6 2.935457000 -1.634060000 0.000133000

6 3.941267000 1.357078000 0.000078000

6 1.718719000 -0.913723000 0.000080000

6 2.548388000 1.532789000 0.000090000

6 1.583052000 0.548653000 0.000053000

1 5.750263000 0.310457000 0.000066000

1 5.001237000 -1.896847000 0.000150000

1 2.830989000 -2.712619000 0.000163000

1 4.517474000 2.273865000 0.000075000

1 2.185599000 2.553056000 0.000133000

6 0.435651000 -1.436444000 0.000088000

1 0.182371000 -2.481750000 0.000112000

6 -0.483837000 -0.357690000 -0.000019000

7 0.253201000 0.802840000 0.000143000

6 -4.046536000 -1.427413000 -0.000001000

6 -2.679788000 -1.477585000 -0.000031000

6 -4.694230000 -0.164734000 0.000040000

1 -2.180378000 -2.437682000 0.000126000

1 -5.776601000 -0.129809000 0.000195000

6 -1.895644000 -0.295520000 -0.000154000

6 -3.984990000 1.003175000 -0.000135000

1 -4.478871000 1.964759000 -0.000055000

6 -2.547649000 1.014304000 -0.000615000

8 -1.898871000 2.105986000 -0.000148000

1 -4.630637000 -2.336282000 0.000152000

1 -0.325042000 1.679528000 0.000166000


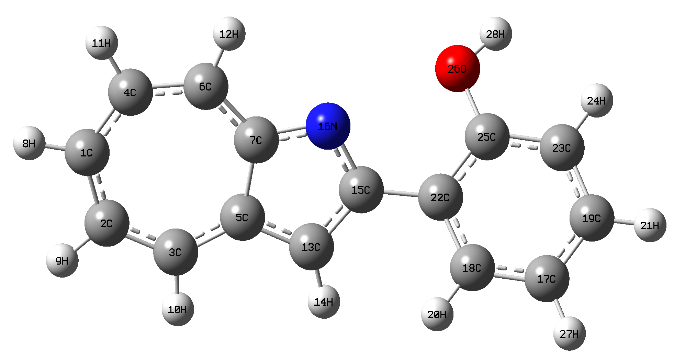


optimized geometries of **R1(O)**

6 4.707966000 0.116312000 -0.063535000

6 4.223183000 -1.122450000 0.365515000

6 2.908744000 -1.547889000 0.494917000

6 4.012817000 1.251246000 -0.467591000

6 1.739699000 -0.835327000 0.230516000

6 2.637085000 1.457514000 -0.547631000

6 1.616802000 0.565802000 -0.244210000

1 5.787673000 0.204905000 -0.083649000

1 4.979678000 -1.851087000 0.629466000

1 2.771628000 -2.565767000 0.843474000

1 4.627523000 2.094520000 -0.757194000

1 2.297930000 2.429433000 -0.884525000

6 0.422297000 -1.270701000 0.360553000

1 0.098233000 -2.237406000 0.708443000

6 -0.397574000 -0.184708000 -0.008883000

7 0.322513000 0.906794000 -0.367786000

6 -3.899471000 -1.476413000 -0.531519000

6 -2.517768000 -1.384462000 -0.456097000

6 -4.672792000 -0.371410000 -0.190821000

1 -1.911682000 -2.233068000 -0.741138000

1 -5.751726000 -0.419160000 -0.245122000

6 -1.866109000 -0.210577000 -0.053600000

6 -4.058227000 0.801055000 0.224177000

1 -4.657844000 1.659874000 0.502446000

6 -2.667837000 0.892431000 0.299616000

8 -2.065633000 2.026096000 0.751785000

1 -4.366439000 -2.393748000 -0.860109000

1 -2.740381000 2.672639000 0.979678000


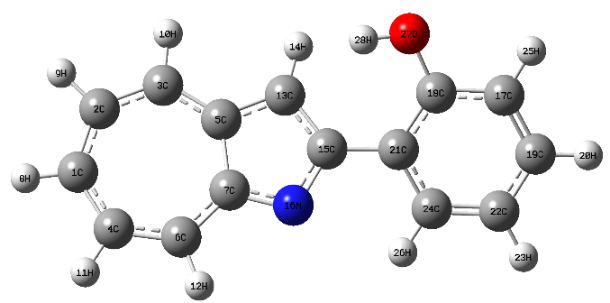


optimized geometries of **R2(O)**

6 -4.712016000 -0.121523000 0.023709000

6 -4.170535000 1.112178000 -0.340670000

6 -2.836471000 1.481505000 -0.454573000

6 -4.068111000 -1.311188000 0.355832000

6 -1.703516000 0.702662000 -0.229330000

6 -2.706158000 -1.587017000 0.411258000

6 -1.643625000 -0.724417000 0.158395000

1 -5.794371000 -0.160318000 0.047611000

1 -4.891463000 1.889297000 -0.562056000

1 -2.652260000 2.507705000 -0.752852000

1 -4.721115000 -2.140079000 0.599177000

1 -2.412751000 -2.593721000 0.682245000

6 -0.364214000 1.085794000 -0.347051000

1 -0.014128000 2.040600000 -0.704989000

6 0.409884000 -0.055890000 -0.044781000

7 -0.367834000 -1.131628000 0.250596000

6 4.109343000 0.732031000 0.197308000

6 2.726490000 0.891746000 0.255550000

6 4.654608000 -0.493394000 -0.150920000

1 5.729493000 -0.604185000 -0.196319000

6 1.869482000 -0.183118000 -0.050155000

6 3.824805000 -1.580589000 -0.426022000

1 4.248781000 -2.539944000 -0.686283000

6 2.451585000 -1.421323000 -0.364943000

1 4.735536000 1.577924000 0.442391000

1 1.790090000 -2.249824000 -0.570643000

8 2.272767000 2.119467000 0.645518000

1 1.325588000 2.073452000 0.821756000


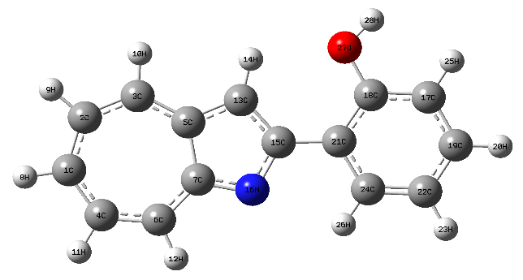


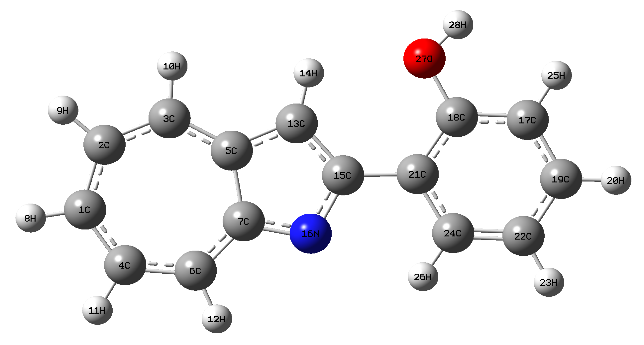


optimized geometries of **R3(O)**

6 -4.726762000 -0.131569000 0.000048000

6 -4.189898000 1.157132000 0.000058000

6 -2.856835000 1.548165000 0.000043000

6 -4.078985000 -1.364673000 0.000020000

6 -1.720042000 0.742328000 0.000014000

6 -2.715460000 -1.640874000 -0.000010000

6 -1.656188000 -0.738512000 -0.000025000

1 -5.809190000 -0.179209000 0.000066000

1 -4.914049000 1.962457000 0.000068000

1 -2.675541000 2.617403000 0.000020000

1 -4.729509000 -2.230563000 0.000032000

1 -2.419193000 -2.682724000 0.000004000

6 -0.385624000 1.150300000 -0.000102000

1 -0.026327000 2.161624000 -0.000173000

6 0.394169000 -0.023256000 0.000000000

7 -0.381078000 -1.146612000 0.000049000

6 4.132593000 0.688362000 -0.000061000

6 2.756811000 0.910810000 -0.000073000

6 4.641178000 -0.601841000 0.000027000

1 5.711561000 -0.755296000 0.000034000

6 1.853317000 -0.169780000 0.000007000

6 3.768732000 -1.686916000 0.000105000

1 4.152367000 -2.697225000 0.000174000

6 2.402552000 -1.463693000 0.000095000

1 4.804425000 1.538917000 -0.000122000

1 1.709962000 -2.291175000 0.000156000

8 2.267601000 2.189844000 -0.000159000

1 3.004885000 2.807421000 -0.000207000


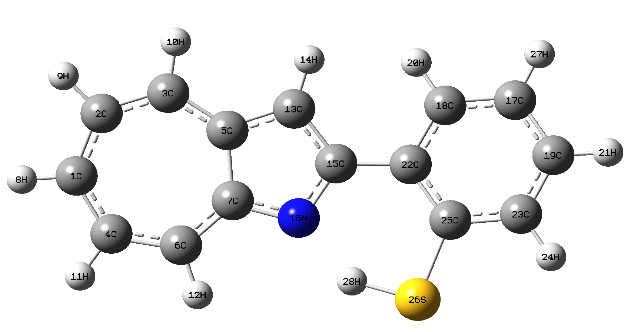


optimized geometries of **Thiol**

6 4.844070000 0.157947000 -0.019714000

6 4.412842000 -1.148790000 0.240098000

6 3.121800000 -1.644108000 0.305863000

6 4.099478000 1.301520000 -0.275553000

6 1.918258000 -0.954263000 0.130418000

6 2.714044000 1.453755000 -0.339195000

6 1.739062000 0.483836000 -0.161918000

1 5.918587000 0.295411000 -0.021096000

1 5.201137000 -1.871362000 0.410835000

1 3.029010000 -2.703114000 0.519731000

1 4.673933000 2.202979000 -0.448383000

1 2.335884000 2.445807000 -0.553180000

6 0.626491000 -1.463866000 0.200050000

1 0.366242000 -2.483346000 0.425089000

6 -0.257710000 -0.386348000 -0.037341000

7 0.425891000 0.769619000 -0.249312000

6 -3.657183000 -1.961405000 -0.239339000

6 -2.292606000 -1.748544000 -0.225903000

6 -4.514602000 -0.873279000 -0.086594000

1 -1.633719000 -2.592788000 -0.363351000

1 -5.586884000 -1.013579000 -0.092833000

6 -1.722964000 -0.469689000 -0.065421000

6 -3.990311000 0.397354000 0.061205000

1 -4.656829000 1.242491000 0.167458000

6 -2.608030000 0.630236000 0.069511000

16 -2.146702000 2.329372000 0.293532000

1 -4.050077000 -2.959243000 -0.373075000

1 -0.827123000 2.133330000 0.040481000


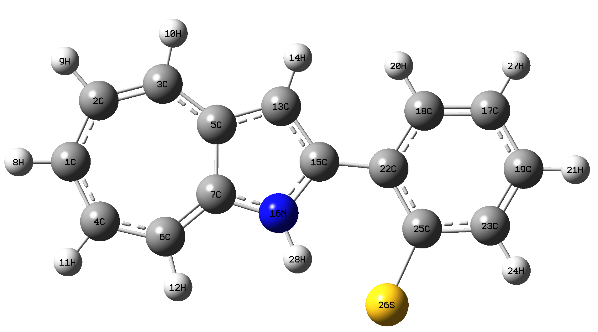


optimized geometries of **Thione**

6 -4.827585000 0.173610000 0.000007000

6 -4.398002000 -1.172184000 0.000018000

6 -3.122568000 -1.680710000 0.000020000

6 -4.075153000 1.328999000 -0.000006000

6 -1.895948000 -0.978896000 0.000010000

6 -2.679073000 1.479987000 -0.000009000

6 -1.734608000 0.476631000 0.000001000

1 -5.901047000 0.313801000 0.000008000

1 -5.193603000 -1.906805000 0.000032000

1 -3.035898000 -2.760849000 0.000042000

1 -4.635640000 2.255289000 -0.000018000

1 -2.297766000 2.493372000 -0.000031000

6 -0.619747000 -1.519080000 0.000043000

1 -0.390965000 -2.569129000 0.000077000

6 0.320954000 -0.458668000 -0.000009000

7 -0.397762000 0.708427000 -0.000032000

6 3.729460000 -1.920274000 0.000015000

6 2.370137000 -1.768642000 0.000007000

6 4.544045000 -0.768574000 0.000011000

1 1.746556000 -2.650723000 0.000026000

1 5.621316000 -0.871248000 0.000031000

6 1.749231000 -0.488550000 -0.000022000

6 3.983717000 0.481666000 -0.000011000

1 4.612480000 1.360000000 -0.000003000

6 2.575873000 0.696252000 -0.000052000

16 2.026503000 2.336391000 -0.000003000

1 4.171509000 -2.906269000 0.000038000

1 0.138951000 1.611923000 -0.000070000


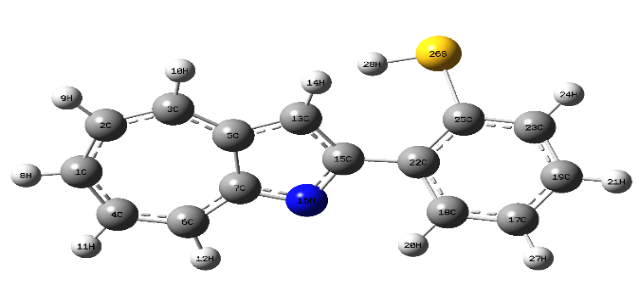


optimized geometries of **R1(S)**

6 -4.812680000 0.185867000 -0.034580000

6 -4.407958000 -1.127294000 0.233012000

6 -3.126218000 -1.647515000 0.306343000

6 -4.046115000 1.314995000 -0.292891000

6 -1.909969000 -0.982122000 0.131699000

6 -2.658135000 1.441253000 -0.351962000

6 -1.701168000 0.454625000 -0.166637000

1 -5.884543000 0.343421000 -0.040563000

1 -5.210762000 -1.833607000 0.404511000

1 -3.055356000 -2.707034000 0.526568000

1 -4.603749000 2.225948000 -0.471331000

1 -2.257338000 2.424129000 -0.566716000

6 -0.626619000 -1.514471000 0.208026000

1 -0.379787000 -2.537439000 0.435039000

6 0.267440000 -0.446172000 -0.029903000

7 -0.384388000 0.719151000 -0.250426000

6 3.718954000 -1.911957000 -0.232765000

6 2.345273000 -1.763041000 -0.203170000

6 4.523582000 -0.781675000 -0.110977000

1 1.718310000 -2.635389000 -0.315680000

1 5.601185000 -0.870061000 -0.133025000

6 1.728584000 -0.508729000 -0.054222000

6 3.944475000 0.467781000 0.032439000

1 4.582389000 1.336002000 0.119007000

6 2.554188000 0.634068000 0.066450000

16 1.886016000 2.275263000 0.284928000

1 4.158859000 -2.891415000 -0.355469000

1 3.083434000 2.813499000 0.586630000


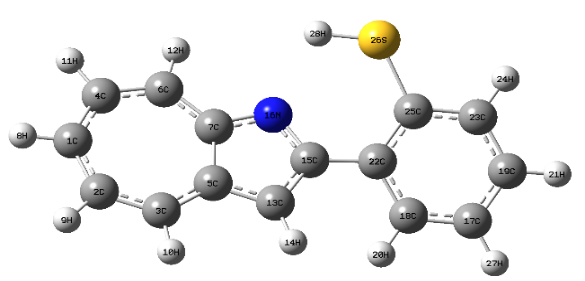


optimized geometries of **R2(S)**

6 4.875835000 -0.060184000 0.013515000

6 4.286027000 1.088422000 -0.520054000

6 2.939327000 1.383436000 -0.681043000

6 4.281414000 -1.214363000 0.514732000

6 1.837258000 0.597510000 -0.347135000

6 2.929783000 -1.533207000 0.616198000

6 1.836181000 -0.760263000 0.244922000

1 5.958935000 -0.051457000 0.037785000

1 4.976241000 1.854358000 -0.851051000

1 2.713645000 2.348574000 -1.121059000

1 4.966860000 -1.973051000 0.871337000

1 2.676919000 -2.500904000 1.031822000

6 0.486262000 0.906593000 -0.512801000

1 0.087156000 1.803678000 -0.952288000

6 -0.240595000 -0.207938000 -0.049394000

7 0.576002000 -1.200330000 0.395554000

6 -3.476315000 -2.056271000 -0.444394000

6 -2.133371000 -1.741504000 -0.327920000

6 -4.430387000 -1.056000000 -0.282946000

1 -1.379813000 -2.505838000 -0.444305000

1 -5.484466000 -1.282521000 -0.366236000

6 -1.695836000 -0.430794000 -0.075021000

6 -4.028145000 0.236207000 0.016904000

1 -4.770453000 1.002496000 0.193241000

6 -2.674124000 0.564514000 0.124150000

16 -2.350885000 2.273959000 0.540568000

1 -3.777293000 -3.072424000 -0.657188000

1 -1.145457000 2.099130000 1.101693000


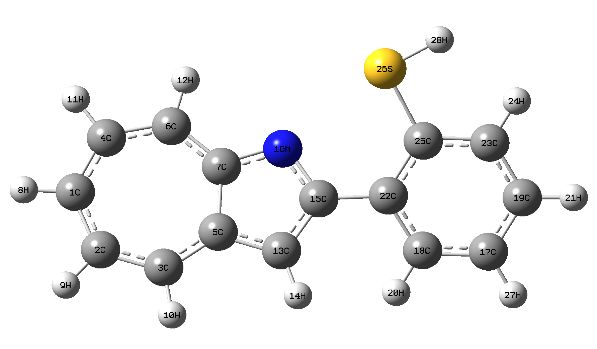


optimized geometries of **R3(S)**

6 4.872507000 -0.057654000 0.035187000

6 4.281132000 1.123801000 -0.416515000

6 2.933388000 1.423676000 -0.566464000

6 4.279701000 -1.248431000 0.447104000

6 1.833736000 0.611037000 -0.299161000

6 2.929771000 -1.578988000 0.516928000

6 1.834137000 -0.785439000 0.195133000

1 5.955395000 -0.046375000 0.067926000

1 4.969672000 1.915502000 -0.684479000

1 2.706080000 2.417616000 -0.935730000

1 4.966560000 -2.028034000 0.752300000

1 2.678712000 -2.574638000 0.861624000

6 0.482303000 0.925533000 -0.453373000

1 0.090589000 1.852236000 -0.833594000

6 -0.242766000 -0.218806000 -0.071188000

7 0.576418000 -1.238324000 0.307433000

6 -3.491949000 -2.065289000 -0.365301000

6 -2.146127000 -1.751473000 -0.301839000

6 -4.435843000 -1.055301000 -0.202952000

1 -1.396985000 -2.519889000 -0.418076000

1 -5.493194000 -1.277383000 -0.250124000

6 -1.696867000 -0.437652000 -0.088440000

6 -4.021680000 0.246620000 0.029586000

1 -4.763570000 1.020193000 0.170424000

6 -2.663946000 0.572654000 0.096109000

16 -2.187017000 2.254182000 0.478930000

1 -3.803782000 -3.085312000 -0.540049000

1 -3.437125000 2.701705000 0.665994000
